# Transcriptomic analysis reveals TME-mediated macrophage IFIT1 upregulation and CX3CR1 suppression drive osteosarcoma progression

**DOI:** 10.3389/fonc.2025.1686854

**Published:** 2025-11-05

**Authors:** Keyi Wang, Huanyang He, Jiamin Liang, Yuangang Su, Jinmin Zhao, Qian Liu

**Affiliations:** Collaborative Innovation Centre of Regenerative Medicine and Medical BioResource Development and Application Co-constructed by the Province and Ministry, Department of Orthopedics Trauma and Hand Surgery, Guangxi Key Laboratory of Regenerative Medicine, The First Affiliated Hospital of Guangxi Medical University, Nanning, China

**Keywords:** osteosarcoma microenvironment, tumor-associated macrophages, transcriptome analysis, IFIT1, CX3CR1

## Abstract

**Introduction:**

Osteosarcoma (OS) is one of the most common bone tumors with an unsatisfactory prognosis for patients. Due to the stagnation in conventional treatments, researchers are exploring therapeutic targets from the tumor microenvironment (TME) and tumor-associated macrophages (TAM). Our study investigates how OS TME influences macrophage gene expression, potentially informing OS treatment strategies.

**Methods:**

RNA sequencing was performed on bone marrow-derived macrophages (BMMs) cultured with or without K7M2 conditional medium (CM) for 48 h to analyze gene expression changes. Single-cell sequencing and PCR were used to examine the expression levels of IFIT1 and CX3CR1. Their functions were verified through flow cytometry, cloning, wound healing, and transwell assays using IFIT1 protein and CX3CR1 inhibitors.

**Results:**

We observed changes in the morphology and transcriptome of BMMs exposed to K7M2 CM. Differentially expressed genes (DEGs) exhibited complex interactions and were enriched in multiple functions and pathways. The upregulation of IFIT1 and the downregulation of CX3CR1 were the most representative. Inhibiting CX3CR1 can promote TAM polarization, thereby accelerating the progression of osteosarcoma. Additionally, increasing IFIT1 also promotes osteosarcoma.

**Conclusions:**

Stimulation of the OS TME can change the gene expression of macrophages. Our findings offer a cellular and molecular reference for future investigations of therapeutic targets of OS.

## 1 Introduction

Osteosarcoma (OS), a highly prevalent primary bone malignancy, predominantly occurs in adolescents and young adults (1). Despite multimodal therapies, the prognosis for patients with OS remains poor due to tumor heterogeneity and complex molecular mechanisms (2, 3). Given the stagnation in conventional treatments, in recent years, researchers have taken interest in seeking potential therapeutic targets from molecular cell signaling components involved in tumor microenvironment (TME) to block OS progression (4, 5). TME is a dynamic network of bone cells, stromal/vascular/immune components, and calcified matrix that hijacks physiological pathways to promote tumor survival and therapy resistance (6, 7). Macrophages are highly plastic myeloid cells that dynamically adapt to microenvironmental cues, regulating tissue homeostasis, inflammation, and host defense through diverse functional phenotypes (8, 9). Macrophages exhibiting tumor infiltration and microenvironmental accumulation are classified as tumor-associated macrophages (TAMs) (10).

Within OS microenvironments, TAMs emerge as the predominant infiltrating immune population, constituting up to 50% of neoplastic cellularity; their density shows clinical correlation with pro-tumorigenic functions (11, 12). Mirroring the investigative prioritization of TME biology, recent OS research has strategically redirected its focus toward TAMs, with the translational objective of developing targeted therapeutic modalities to enhance clinical outcomes in OS patients (13). However, the molecular mechanism to explain the influence of TME on macrophages is still poorly understood. Transcriptome analysis enables comprehensive and rapid acquisition of sequence and expression information for nearly all transcripts in a specific cell or tissue under a given condition, including protein-coding mRNAs and various non-coding RNAs (14). By analyzing transcript structure and expression levels, it can reveal critical biological questions such as gene expression differences, structural variations, and molecular (15).

In this study, bone marrow-derived macrophages (BMMs) were grown in a complete α-MEM medium or a conditional medium (CM) of K7M2 cells for 48 h. Then, we performed RNA sequencing to explore the gene expression changes. We found that the profiles of differentially expressed genes (DEGs) functionally engaged with multiple oncogenic pathways and exhibited significant crosstalk with TME. The upregulation of IFIT1 and the downregulation of CX3CR1 are the most representative. These results may promote the understanding of the roles of macrophages in TME and provide molecular evidence for novel therapeutic targets of OS.

## 2 Materials and methods

### 2.1 Media and reagents

OS cell line K7M2 was kindly provided by Shanghai Whelab Bioscience Limited.α-MEM, and DMEM and fetal bovine serum (FBS) were all from Gibco (MD, USA). M-CSF was purchased from the R&D Biotechnology (Minneapolis, MN, USA). Penicillin/streptomycin (PS), TRIzol^®^ lysate, and RevertAid™ reverse transcriptase kits were sourced from Thermo Fisher Scientific (Scoresby, VIC, Australia). Qubit™ RNA assay kit was from Life Technologies (Waltham, MA, USA).

### 2.2 OS cell line culture

K7M2 cells were propagated in DMEM (10% FBS, 37°C, 5% CO_2_/95% air, >95% humidity). The CM of K7M2 (2.5 × 10^5^ cell/mL) was collected and used for further experiments.

### 2.3 Isolation of BMMs

C57BL6/J mice were used at 6 weeks of age. Following epiphyseal osteotomy of murine femora and tibiae, the bone marrow was perfused with α-MEM. The cellular suspension underwent differential centrifugation prior to resuspension in complete medium (α-MEM with 10% FBS, 1% PS, and 25 ng/mL M-CSF) for T75 priming.

### 2.4 K7M2 CM intervention

The complete α-MEM medium was renewed every 2 days until the BMMs reached approximately 95% growth. Following enzymatic dissociation and differential centrifugation, cellular was seeded in 6-well plates (2.5 × 10^5^ cells/well). The complete α-MEM medium or α-MEM medium containing 20% K7M2 CM was used to culture BMMs respectively for 48 h.

### 2.5 CCK8 assay

Treated cells in 96-well plates were incubated for 48 h. CCK8 reagent (10 μL/well) was then added and incubated for 3 h, and absorbance was measured at 450 nm.

### 2.6 Immunofluorescence

BMMs were fixed with 4% paraformaldehyde, permeabilized using 0.1% Triton X-100, and blocked with 5% BSA. Anti-CD206 and anti-CD163 antibody (Zenbio, Chengdu, China) were incubated overnight at 4°C, followed by secondary antibody and Diamidino-2-phenylindole dihydrochloride (DAPI) counterstaining.

### 2.7 RNA extraction

Following RNA isolation from BMMs, conditioned with K7M2 secretome and untreated counterparts using the TRIzol™ reagent, RNA integrity and concentration were quantified via Qubit™ 2.0 fluorometer with the corresponding RNA assay kit.

### 2.8 RNA-sequencing and transcriptome analysis

RNA-seq libraries were assembled with Hieff NGS™ MaxUp Dual-mode mRNA Library Prep Kit (Yeasen, Shanghai, China), followed by DNBSEQ-T7 platform (MGI Tech, Shenzhen, China) sequencing (n = 3).

Sequencing datasets underwent quality control processing with Trimmomatic (v0.36) and FastQC (v0.11.2) for adapter trimming and read visualization. A representative subset of 10,000 high-quality reads was randomly subsampled for taxonomic profiling using BLASTN against the NCBI NT database. Genome-guided alignment was subsequently executed via HISAT2 (v2.1.0) with GRCh38 reference genome, followed by alignment quality metrics computation using RSeQC (v2.6.1). Gene expression quantification was conducted via transcripts per million (TPM) normalization. Differential expression profiling employed DESeq2 (v1.12.4) with stringent thresholds (FDR <0.05, absolute log2FC >2). Significantly altered genes underwent functional annotation through STRING database (v11.5) for protein–protein interaction networks (PPI) reconstruction; topGO (v2.24.0) for gene ontology (GO) enrichment and clusterProfiler (v3.0.5) for EuKaryotic Ortholog Groups (KOG) and KEGG analysis.

### 2.9 Single-cell RNA sequencing analyses

GSE162454 (16, 17) scRNA-seq data (.h5) and annotations were obtained from Tumor Immune Single-Cell Hub (TISCH), processed with MAESTRO/Seurat in R, and re-clustered via t-SNE.

### 2.10 Real-time PCR

Transcript levels of *Ifit1* and *Cx3cr1* were quantified via SYBR green-based RT-PCR. Normalization employed endogenous β-actin, with primer sequences detailed in **Table 1**.

**Table 1:** The primer sequences.

Gene	Forward (5’-3’)	Reverse (5’-3’)
*Ifit1*	CTCCACTTTCAGAGCCTTCG	TGCTGAGATGGACTGTGAGG
*Cx3cr1*	GAGTATGACGATTCTGCTGAGG	CAGACCGAACGTGAAGACGAG
*Actb*	TCCTCCCTGGAGAAGAGCTA	ATCTCCTTCTGCATCCTGTC

### 2.11 Flow cytometry

TAMs were washed with PBS, stained with anti-CD206 and anti-CD86 antibody (Thermo Fisher, MD, USA) for 30 min and then analyzed by flow cytometry (BD Accuri C6 Plus).

### 2.12 Wound-healing assay

The scratch insert was removed following K7M2 cells (10^4^ per well) attachment. During treatment of BMMs with K7M2 CM, JMS-17-2 (0.25nM, MedChem, Express New Jersey, USA) was either added or omitted. After 48 h, the medium was replaced with complete α-MEM. Following a subsequent 48-h culture period, supernatants were collected as CM (JMS-17–2 free) and iCM (JMS-17–2 supplemented). K7M2 cells were cultured in DMEM (1% FBS) supplemented with 20% CM, iCM or 10 ng/mL IFIT1 (Cusabio, Wuhan, China). Images were captured at 0/48/72 h.

### 2.13 Transwell assay

K7M2 cells (10^4^ per well) were seeded in serum-free medium atop a matrigel-coated (invasion) or uncoated (migration) transwell insert. Migrated/invaded cells on the lower membrane were fixed and stained (crystal violet) after 48 h.

### 2.14 Clinical database analysis

We have extracted the gene expression profiles and clinical data of OS patients from TARGET (https://ocg.cancer.gov/programs/target). Kaplan–Meier curves were generated using the survival package in R software. The correlation plot for multiple genes was displayed using the pheatmap package in R software (version 4.0.3). We extracted data in TPM format from the TCGA database (https://portal.gdc.cancer.gov). We performed univariate Cox proportional hazards regression analyses and used the forest-plot package to generate a forest plot. Additionally, the nomogram was constructed using the rms package.

## 3 Results

### 3.1 Morphological alterations and M2 polarization of BMMs following co-culture with K7M2 CM

**Figure 1:**
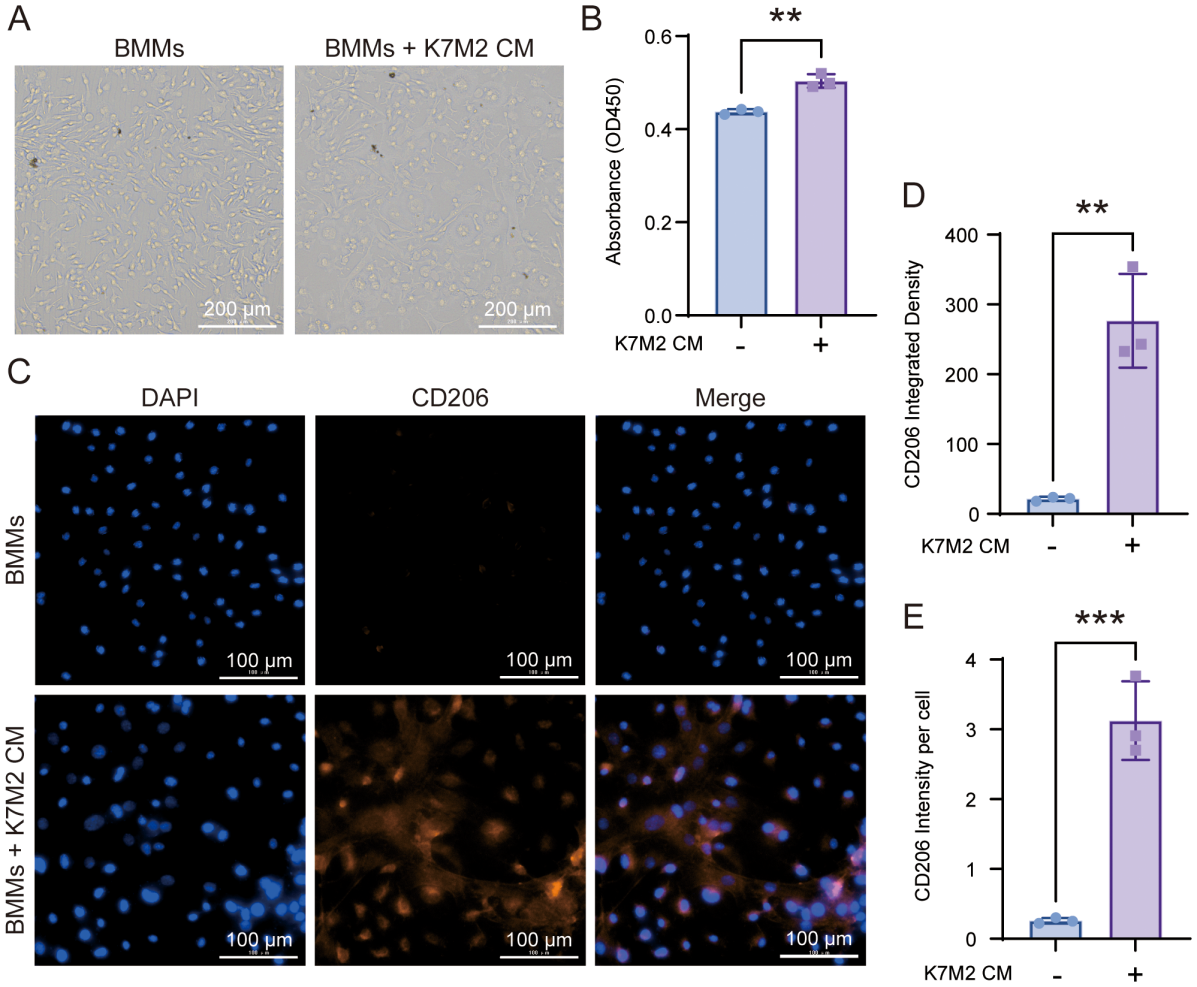
The morphology of bone marrow-derived macrophage (BMM) changes after co-culture with K7M2 conditional medium (CM). **(A)** Representative image in different groups after being fixed with 4% paraformaldehyde (PFA). Scale bar, 100 μm. **(B)** Cell viability of BMMs following a 48-h treatment with or without K7M2 CM, assessed by CCK-8 assay. **(C)** Representative immunofluorescence images of CD206-positive cells stimulated with or without K7M2 CM. **(D–E)** Quantification of CD206 immunofluorescence intensity. * p < 0.05, ** p < 0.01, *** p < 0.001.

To observe the effect of OS TME on macrophages, we co-incubated BMMs with K7M2 CM. As shown in **Figure 1**, there were obvious changes in the morphology of tested BMMs. The BMMs exposed to K7M2 CM grew far from one another in whirling arrays and presented with flat, elliptical or amorphous shape, which was different from the slender and fusiform shape of the control group. CD206 is a marker of pro-tumor M2 macrophage (18). CD163^+^ M2 macrophages also correlate with tumor progression (19). The immunofluorescence results showed that under stimulation by K7M2 CM, BMMs change toward M2-type TAM (**Figures 1C, D** and **Supplementary Figure S1A, B**).

### 3.2 Overview of transcriptome sequencing data

RNA-seq raw data underwent rigorous quality control. High-confidence clean reads demonstrated Q30 scores exceeding 97% across biological replicates, with genomic ‌Guanine-Cytosine (GC) content stabilized from 51.65% to 52.39% (**Table 2**). Alignment to the Mus musculus reference genome achieved 99% total mapping efficiency, including 94.8% to 95.17% uniquely aligned reads (**Table 3**). The raw data employed in the analysis demonstrated adequate quality.

**Table 2:** Quality control of the sequencing data.

Sample	Total reads count	Total bases count (bp)	Clean reads count	Clean bases count (bp)	Clean Q30 (%)	Clean GC (%)
C1	64546500	9681975000	62441134	9016123502	97.59%	52.39%
C2	65956542	9893481300	64394164	9299028201	97.84%	52.15%
C3	55837134	8375570100	53812340	7854563724	97.37%	52.05%
P1	64962148	9744322200	63060392	9174214765	97.76%	51.70%
P2	58170278	8725541700	55983736	8176019241	97.35%	51.65%
P3	62170498	9325574700	60055868	8706097773	97.49%	51.95%

**Table 3:** Reads number of Mus musculus and the summary of mapped data.

Sample	Total reads	Total mapped	Multiple mapped	Uniquely mapped	Mus musculus reads number (‱)
C1	61285544 (100.00%)	60852808 (99.29%)	2588071 (4.22%)	58264737 (95.07%)	8333
C2	62237802 (100.00%)	61802307 (99.30%)	2801468 (4.50%)	59000839 (94.80%)	8191
C3	53020786 (100.00%)	52730524 (99.45%)	2282296 (4.30%)	50448228 (95.15%)	8367
P1	62342316 (100.00%)	61958998 (99.39%)	2736798 (4.39%)	59222200 (95.00%)	8572
P2	55027012 (100.00%)	54699722 (99.41%)	2329120 (4.23%)	52370602 (95.17%)	8473
P3	59412940 (100.00%)	59060504 (99.41%)	2700507 (4.55%)	56359997 (94.86%)	8583

### 3.3 DEGs analysis and construction of PPI network

**Figure 2:**
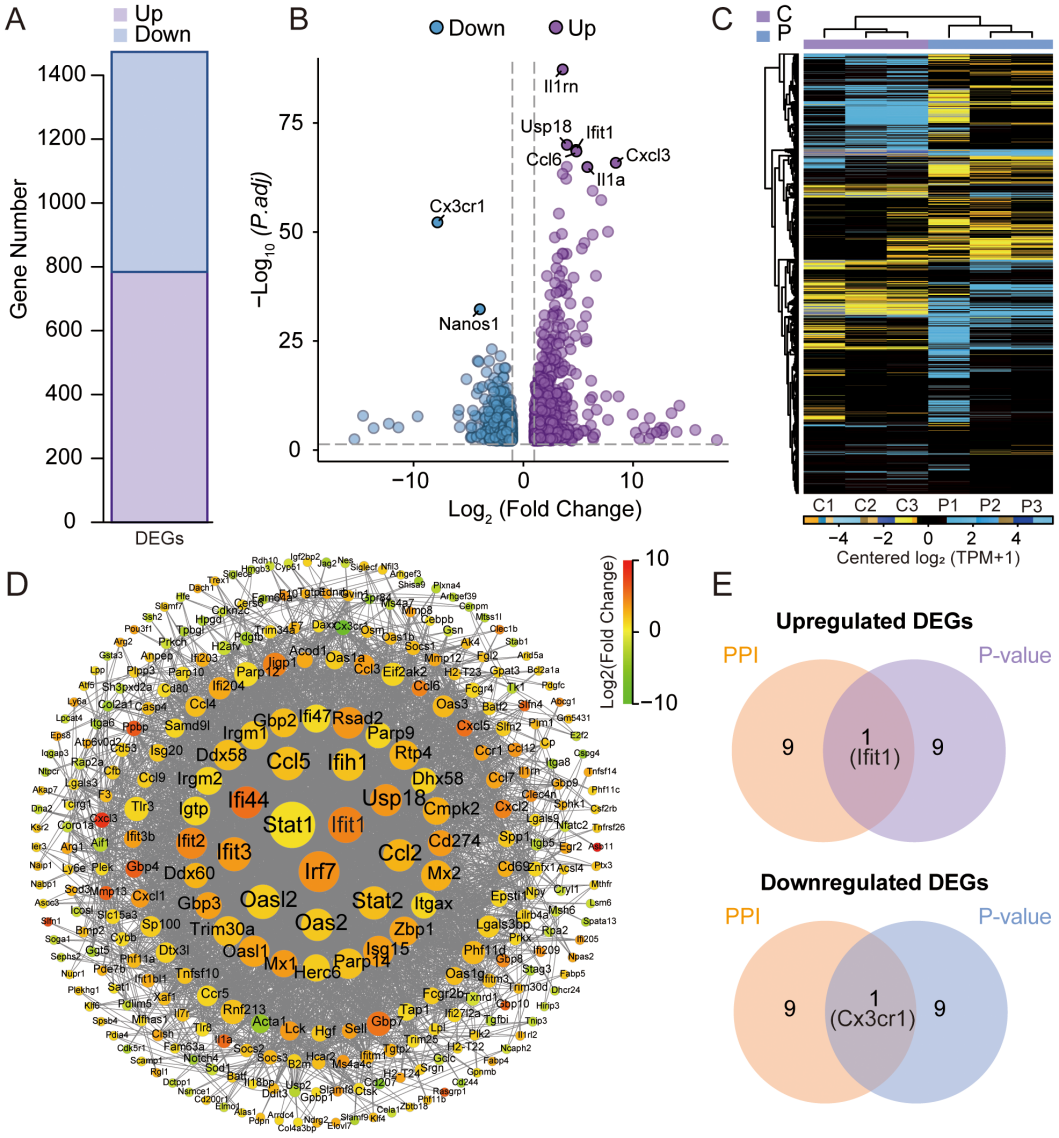
Analysis of differentially expressed genes (DEGs) by RNA sequencing. **(A)** Statistical bar chart of the differential expression analysis. **(B)** Volcano plots for DEGs between the K7M2 CM co-culture and control groups. **(C)** Clustering heatmap of DEGs. **(D)** Comparative Analysis of protein–protein interaction (PPI) networks. **(E)** Venn diagram for the upregulated and downregulated genes.

RNA-seq differential expression profiling revealed 1,473 significantly dysregulated transcripts in the experimental cohort. Among these DEGs, 784 were upregulated and 689 were downregulated in BMMs exposed to K7M2 CM as visualized in **Figure 2A**. The volcano diagram was conducted for the differential expression trend and the DEGs distributions between different groups were analyzed using the hierarchical clustering heatmap method (**Figures 2B, C**). **Figure 2D** shows the PPI network of DEGs. DEGs were represented as nodes whose size was proportional to the degree, and the interactions between DEGs were depicted as edges. Following intersection analysis of upregulated/downregulated gene subsets, a dual-criteria selection framework identified overlapping candidates between the top 10 most significantly DEGs and the hub genes with maximal node degrees in the PPI network. This integrative approach revealed Ifit1 and Cx3cr1 as consensus biomarkers (**Figure 2E**).

### 3.4 GO and KOG enrichment analysis

**Figure 3:**
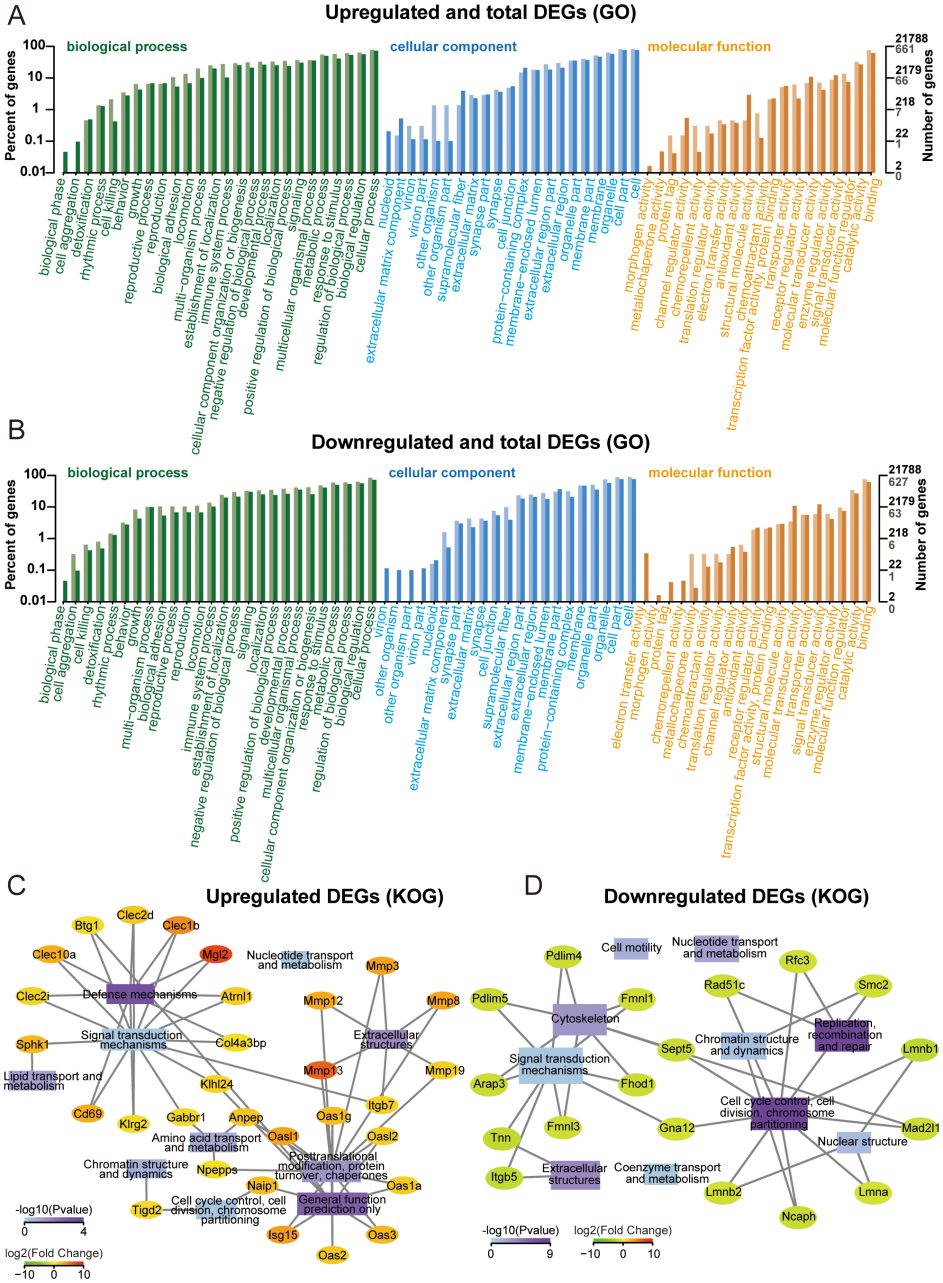
Gene ontology (GO) and KOG enrichment analysis. **(A)** GO annotation classification bar plot of upregulated genes. **(B)** GO annotation classification bar plot of downregulated genes. **(C)** Significantly enriched functional interaction network of upregulated genes. **(D)** Significantly enriched functional interaction network of downregulated genes.

Multi-dimensional functional annotation GO, KOG, and KEGG pathways was systematically implemented to delineate the biological significance of DEGs. As shown in **Figures 3A, B**, functional classification was displayed on the horizontal axis, DEGs were represented by light colored columns, while the total genes were represented by the dark ones. The functional annotation demonstrated enrichment patterns of up and down-regulated DEGs across GO categories. KOG analysis was performed on upregulated and downregulated DEGs, only the top 10 functions with the highest enrichment were displayed. Transcriptional profiling revealed 14 upregulated DEGs demonstrating statistically significant associations with “signal transduction mechanisms” and “post-translational modification, protein turnover, and chaperones” (**Figure 3C**). As for downregulated DEGs, 10 DEGs participated in “cell cycle control, cell division, chromosome partitioning”, and 9 participated in “signal transduction mechanisms” (**Figure 3D**). GO and KOG analyses provide directional guidance for further in-depth understanding of gene functions and biological processes.

### 3.5 KEGG enrichment analysis

**Figure 4:**
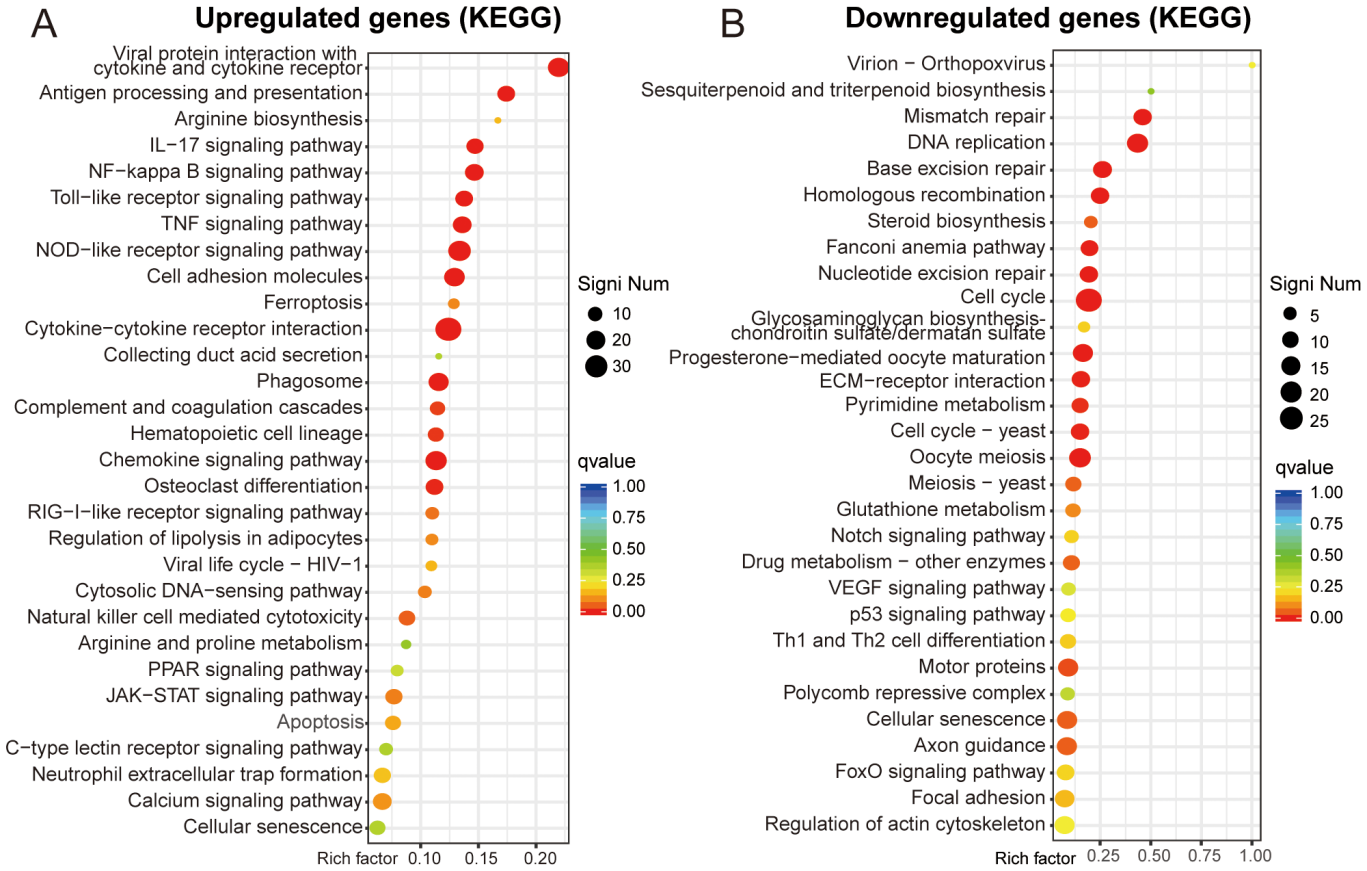
KEGG enrichment analysis. **(A, B)** Functional enrichment scatter plot of upregulated and downregulated genes.

KEGG pathway annotation identified significant enrichment of upregulated DEGs in “viral protein interaction with cytokine and cytokine receptor” and “antigen processing and presentation”. At the same time, the downregulated DEGs were mostly related to “mismatch repair” and “DNA replication” (**Figures 4A, B**). KEGG analysis facilitates the identification of genes involved in the pathogenesis of OS. Additionally, it enables the mapping of these genes to specific metabolic pathways and serves as a basis for drug and material design, thereby facilitating clinical translation (20).

### 3.6 Expression of *Ifit1* and *Cx3cr1*

**Figure 5:**
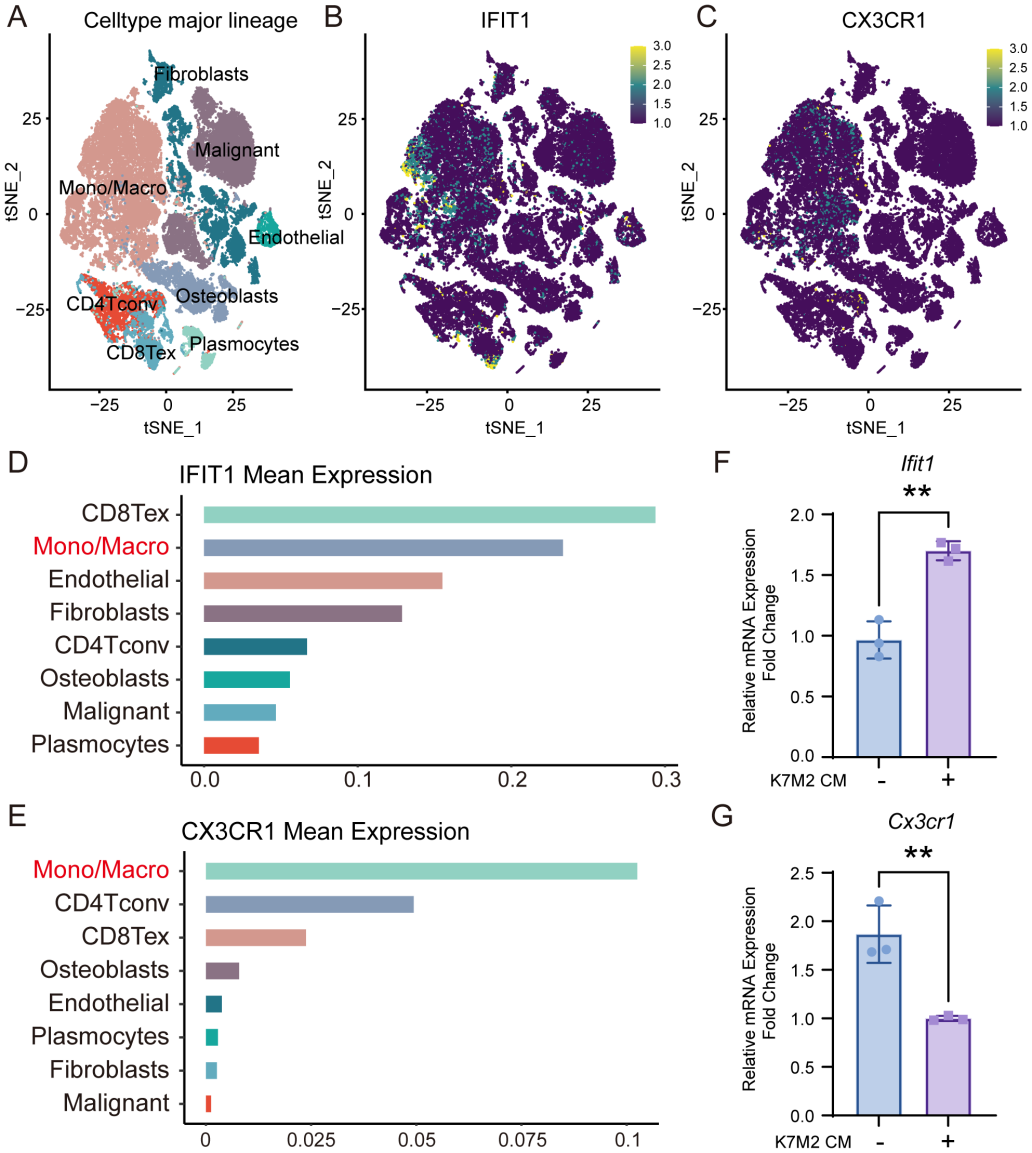
The expression of IFIT1 and CX3CR1 in OS. **(A)** The t-SNE plot of single-cell clustering. **(B, C)** The t-SNE plot of the expression distribution of IFIT and CX3CR1 in different cells. **(D, E**) The bar chart of the expression abundance of IFIT and CX3CR1 in different cells. **(F, G)** Relative expression levels of Ifit1 and Cx3cr1 in BMMs with or without K7M2 CM, normalized to β-actin.

To investigate the specific expression profiles of Ifit1 and Cx3cr1 genes, we analyzed scRNA-seq data from tissues of patients with primary OS (**Figure 5A**). Both IFIT1 (**Figures 5B, D**) and CX3CR1 (**Figures 5C, E**) exhibited high expression in monocyte/macrophage subpopulations, aligning with prior BMM sequencing outcomes. The expression levels of IFIT1 and CX3CR3 also influenced patient clinical outcomes. Patients with high IFIT1 expression and low CX3CR3 expression exhibited poorer prognosis, with a higher cumulative risk of adverse clinical events (**Supplementary Figure S2A, B**). We constructed a forest plot based on the results of Cox proportional hazards regression analysis and generated a nomogram to investigate the association between IFIT1 and CX3CR1 expression levels and clinical prognosis (**Supplementary Figure S2D, E**). PCR assessed the expression levels of Ifit1 and Cx3cr1, revealing that upon intervention with K7M2 CM, Ifit1 expression was significantly upregulated, whereas Cx3cr1 expression was downregulated (**Figures 5F, G**).

### 3.7 Function of IFIT1 and CX3CR1

**Figure 6:**
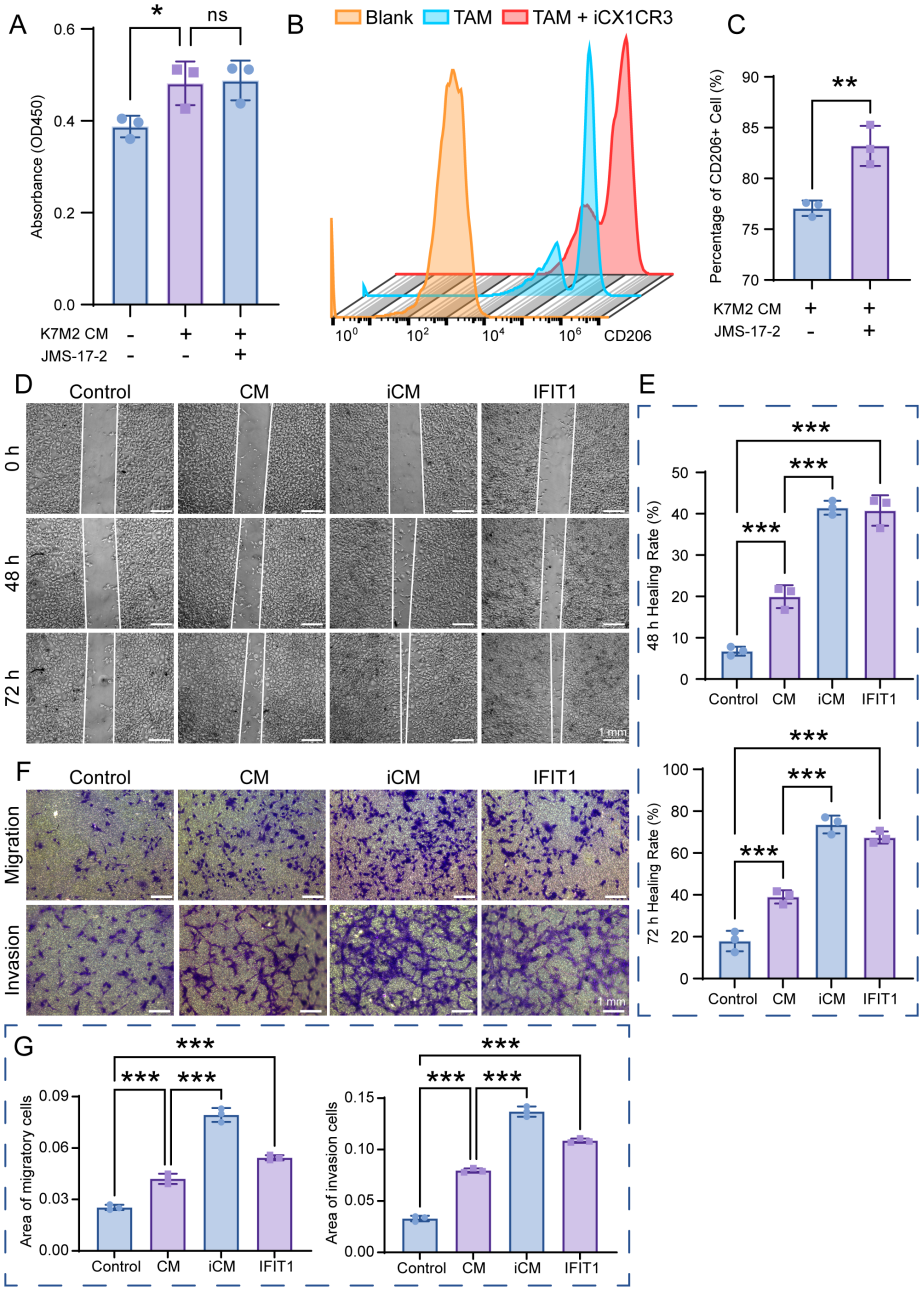
The function of IFIT1 and CX3CR1 in osteosarcoma (OS). **(A)** Cell viability of BMMs after a 48-h treatment with or without K7M2 CM and JMS-17-2, assessed by CCK-8 assay. **(B, C)** TAMs treated with or without JMS-17–2 for 48 h were stained with antibodies against CD206 and analyzed using flow cytometry. **(D)** Wound-healing assay to assess the migration capabilities of K7M2 cells. **(E)** Quantification of the K7M2 healing rate. **(F)** Transwell assay to assess the migration and invasion capabilities of K7M2 cells. **(G)** Quantification of migratory and invasive cell areas. * p < 0.05, ** p < 0.01, *** p < 0.001. ns, no significance, p > 0.05.

We employed a CX3CR1 inhibitor (JMS-17-2) and the IFIT1 protein to investigate the functions of CX3CR1 and IFIT1 in the OS TME. CCK-8 assays revealed that BMMs exhibited slight proliferation in K7M2 CM, while JMS-17–2 showed no cytotoxicity toward BMMs. Furthermore, CX3CR1 inhibition did not suppress alterations in cell viability induced by TME (**Figure 6A**). Flow cytometry results demonstrated that K7M2 CM promoted M2 polarization of BMMs, and JMS-17–2 further enhanced the generation of M2-type TAMs (**Figures 6B, C** and **Supplementary Figure S1C–E**). These findings suggest that the CX3CR1 receptor may functionally suppress M2 polarization of TAMs. CM were collected from the control group BMMs (2.5 × 10^5^ cell/mL), while iCM were collected from JMS-17-2-treated BMMs (2.5 × 10^5^ cell/mL), both of which were induced by K7M2 CM, to investigate the impact of Cx3cr1 downregulation on OS. Following stimulation with K7M2 CM, BMMs in turn promoted the migration and invasion of K7M2 cells. Furthermore, the suppression of CX3CR1 expression amplified the promoting effects of TAMs on K7M2 cells. Compared with the control group, K7M2 cells subjected to IFIT1 intervention exhibited enhanced migratory and invasive capabilities, suggesting that the upregulation of IFIT1 expression also exerts a pro-tumorigenic effect (**Figures 6D–G**).

## 4 Discussion

TME-mediated immunotherapy brings new possibilities for the treatment of OS (21). TME has become a central focus of oncology due to its multifaceted involvement in oncogenesis, disease evolution, metastatic dissemination, and therapeutic recalcitrance (22). As pivotal effectors in tumor immunology, macrophages can be recruited to TME and induce tumoricidal immune responses through cytokines and/or chemokines. The application of macrophages for anti-tumor delivery is regarded as one of the most promising methods (23, 24). Previously, researchers have shown that cytokines and bacterial products drive macrophage ontogeny and phenotypic polarization, which effectively prevent tumor progression and enhance survival in mice (25). However, macrophages do not only have a positive killing effect on tumors, but a double-edged sword relationship (26). TAMs represent the most abundant immune subset in the TME, displaying a functional continuum from tumor-suppressive to tumor-promoting states (27). The functional plasticity of TAMs represents a potential therapeutic target in cancer treatment, while posing mechanistic and translational challenges (28). Exploring the impact of TME on the pro-tumorigenic and anti-tumorigenic features of TAMs is of great significance for exerting their plasticity in the treatment of OS.

In this study, BMMs were cultured in K7M2 CM for 48 h followed by transcriptomic profiling to explore the gene expression changes. The analysis of DEGs focuses on Ifit1 and Cx3cr1 (**Figure 2**). IFIT1 is a member of the interferon-induced protein with tetratricopeptide repeats (IFIT) genes family (29). Although the antiviral mechanisms of IFIT proteins have been extensively characterized, recent studies implicated that they may also serve as critical regulators in oncogenesis (30). Li et al. (31) found that IFIT1 can enhance pancreatic cancer cell proliferation, migration, and invasion while modulating epithelial–mesenchymal transition through Wnt/β-catenin pathway. Liu et al. (32) demonstrated that IFIT1 silencing suppressed the IL-17/IL-1β expression and attenuated hepatocellular carcinoma cell migration. Additionally, the cancer-promoting effects of IFIT1 have been reported in colorectal cancer, oral squamous cell carcinoma, and nasopharyngeal carcinoma (33, 34l 35). Our experimental results demonstrate that the TME upregulates Ifit1 expression in BMMs, and the IFIT1 protein in turn promotes OS invasion and migration (**Figures 6D–G**). Moreover, the role of IFIT1 may be closely related to STAT1 and β-catenin (**Supplementary Figure S3**). The inhibition of IFIT1 may serve as a therapeutic target to impede OS progression. The CX3CL1-CX3CR1 signaling axis, formed by a ligand–receptor interaction between transmembrane CX3CL1 and its cognate receptor, modulates malignant phenotypes encompassing proliferation, migration, invasion, and apoptosis resistance in cancer, suggesting its therapeutic relevance (36). Studies have demonstrated that the inhibition of CX3CR1 promotes macrophage polarization toward the M2 phenotype, which may be associated with reduced levels of pro-inflammatory cytokines (e.g., TNF-α, IL-6) and the suppression of NF-κB signaling (37, 38). Meanwhile, some researchers have reached opposite conclusions, and these reports both implicated another chemokine (CCL2) or chemokine receptor (CCRL2) in addition to CX3CR1. (39, 40). M2 macrophage polarization is associated with IL-4 and IL-13 (41). Studies have reported that increased CX3CR1 expression correlates with reduced IL-4 and IL-13 levels (42). We hypothesize that CX3CR1 inhibition may promote M2 polarization by enhancing their secretion. As for skeletal-related diseases, CX3CR1 has been implicated in rheumatoid arthritis (RA), osteoarthritis (OA), intervertebral disc degeneration (IDD), and bone metastasis of cancer, primarily through its roles in adhering to fibroblast-like synoviocytes (FLSs), endothelial cells, cancer cells, enhancing osteoclastogenesis, osteogenesis, and promoting inflammatory responses (43–47). However, most of these studies concentrate on inflammation and do not extend to tumors. TME sustains malignancy through hypoxia, reduced pH, and elevated interstitial fluid pressure (48, 49). CX3CR1 binds to CX3CL1 to mediate adhesion and chemotaxis in TME (50). The infiltration of M2-polarized TAMs serves as a critical driver of malignant tumor progression (51–53). Our results demonstrated that the inhibition of CX3CR1 promoted M2 polarization of macrophages within the TME, while simultaneously augmenting the pro-tumor effects of M2-type TAMs on OS (**Figures 6B, C**). Based on the analysis of clinical data and our own sequencing data, we hypothesize that CX3CR1 may promote M2 polarization by modulating the NFKB1 (**Supplementary Figure S3**). Reports indicate that CX3CR1 exhibits both tumor-promoting and tumor-suppressing effects in oncology, potentially linked to tumor heterogeneity and immune system complexity (54–58). The function of CX3CR1 in the OS TME is regulated by hypoxia, bone matrix remodeling, and the immune microenvironment. Its dual roles (pro-tumorigenic and anti-tumorigenic) depend on the form of CX3CL1 (membrane-bound FKN and soluble FKN) and local protease activity (ADAM10/17) (55). The therapeutic targeting of CX3CR1 for OS requires further experimental validation and mechanistic investigation. The GO enrichment analysis shows that K7M2 CM exposure impacts the cell, binding, and cellular process of BMMs. As for the KOG analysis, the enrichment network further elucidates the interaction between functions and DEGs (**Figure 3**). The sequencing data indicated genes involved in interaction with cytokine-cytokine receptor, antigen processing and presentation, and mismatch repair (MMR) pathways according to the KEGG enrichment analysis (**Figure 4**). Cytokines exert complex and significant effects on tumors (59). Emerging evidence indicates that blockade of the CCL2-CCR2 axis suppresses monocyte infiltration and exhibits therapeutic efficacy in metastatic osteosarcoma (60). Liao et al. (61) found that CCL3 stimulates vascular endothelial growth factor and angiogenesis in OS by JNK, ERK, and p38 phosphorylation. In the analysis of Tsai et al. (62), they identified that CCL4 stimulates integrin αvβ3 expression and OS cell migration via FAK/AKT/HIF-1α pathways. Other enriched chemokines are also considered as diagnostic or therapeutic genes of OS such as CCL5, CXCL1, CXCL2, CXCL5, and CXCL6 (63–66). The differentiation of TAM subsets is involved in antigen processing and presentation. Antitumorigenic TAMs retain antigen-presenting cell features including elevated MHCII expression, phagocytic/tumoricidal activity, and pro-inflammatory cytokine secretion to activate adaptive immunity, whereas pro-tumorigenic TAMs exhibit immunosuppression via diminished MHCII and upregulated inhibitory molecules (67). MMR has an implication for immunosurveillance and immunotherapy, which is relevant to tumor mutational burden and immune checkpoint blockade (68).

## 5 Conclusions

This study is primarily based on murine sequencing data and public databases, and the expression patterns and prognostic value of IFIT1 and CX3CR1 have not yet been comprehensively validated in clinical samples. Although the roles of key factors such as STAT1 and NF-κB have been preliminarily revealed, the specific mechanisms require further experimental investigation. These limitations do not undermine the scientific validity and innovation of the current findings; instead, they provide clear directions for future research: expanding clinical sample validation (using human OS tissues), and in-depth exploration of signaling pathway regulatory mechanisms (using overexpression/knockdown models of IFIT1 and CX3CR1). In summary, TME stimulation can cause changes in the gene expression of macrophages. Understanding the function of DEGs would help further enable systematic dissection of OS pathogenesis across molecular hierarchies. IFIT1 and CX3CR1 may merge as potential therapeutic targets for OS.

## Data Availability

The datasets presented in this study can be found in online repositories. The names of the repository/repositories and accession number(s) can be found below:https://www.ncbi.nlm.nih.gov/, GSE305592.
