# Supplementary material for: Transcriptomic analysis reveals TME-mediated macrophage IFIT1 upregulation and CX3CR1 suppression drive osteosarcoma progression
Source: Front Oncol. 2025 Nov 5;15:1686854. doi: 10.3389/fonc.2025.1686854 (PMC12626788; doi:10.3389/fonc.2025.1686854)
Supplement: Supplementary file 1 [file DataSheet1.docx]

**
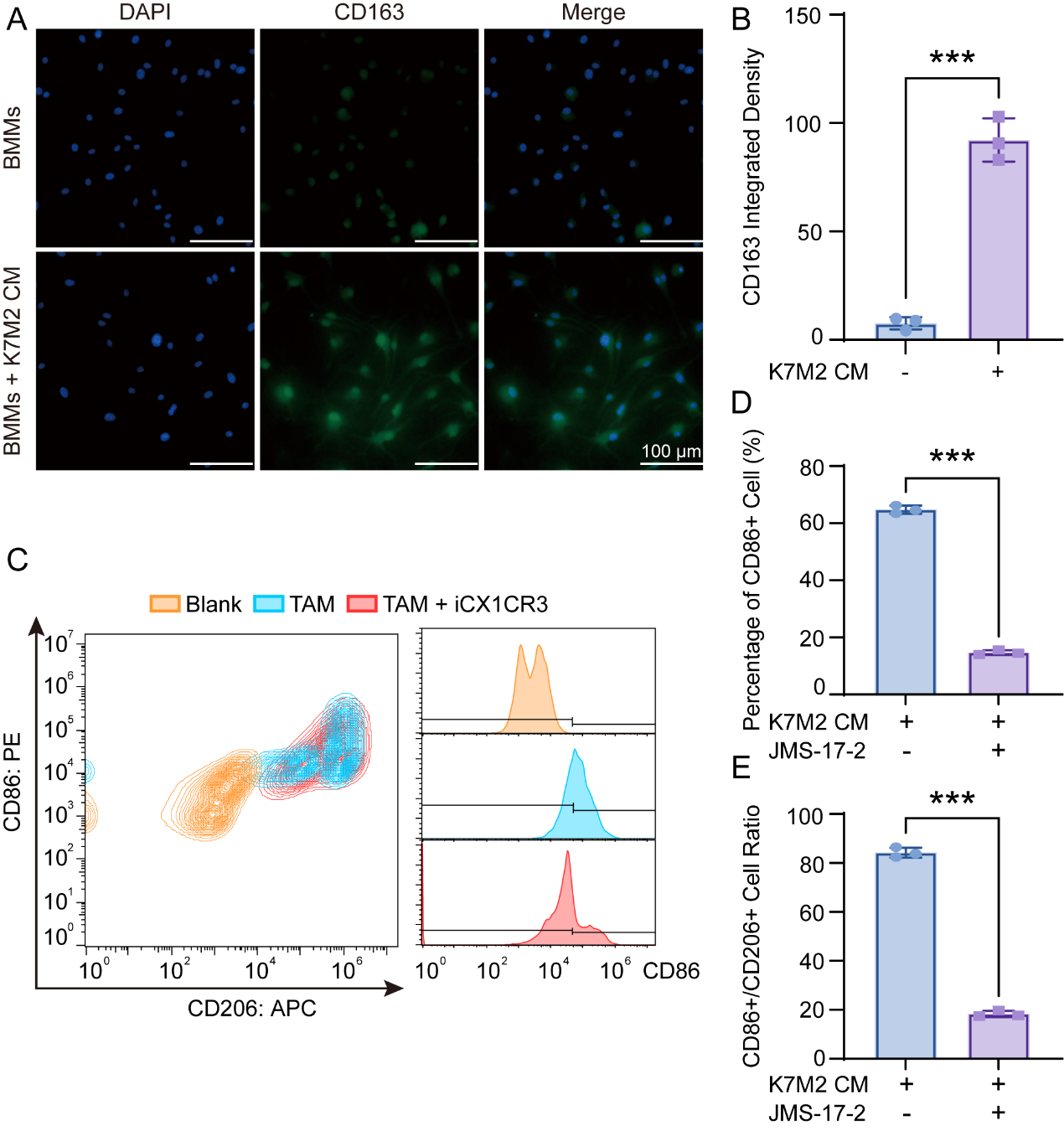
**

**Fig. S1. K7M2 CM and the expression of CX3CR1 influence the polarization of BMMs.** (A) Representative immunofluorescence images of CD163^+^ cells stimulated with or without K7M2 CM‌. (B) Quantification of CD163 Immunofluorescence Intensity. （C-E）TAMs treated with or without JMS-17-2 for 48 hours were stained with antibodies against CD206, CD86 and analyzed using flow cytometry.

**
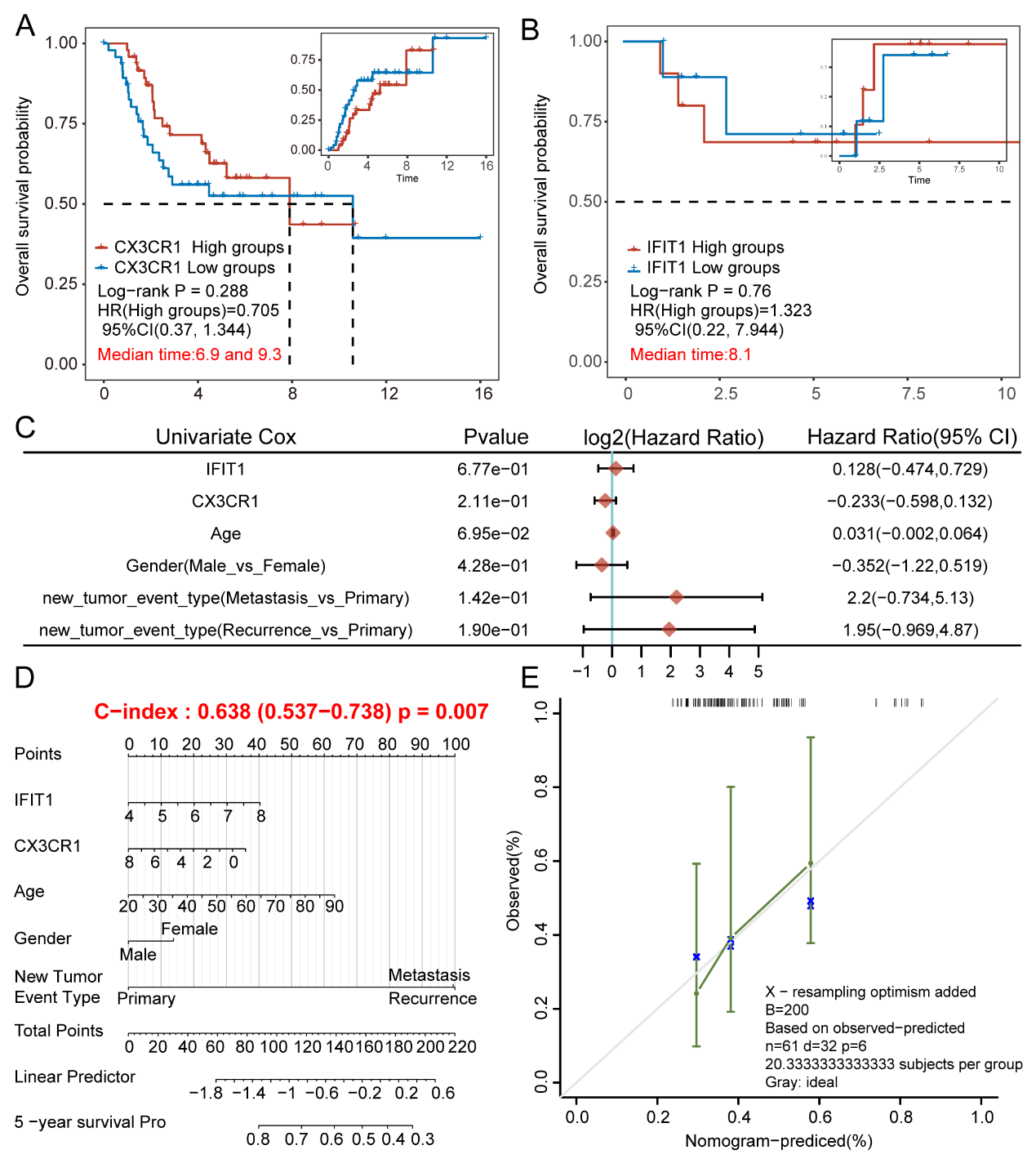
**

**Fig. S2. Association between IFIT1/CX3CR1 expression levels and clinical prognosis.** (A-B) The KM survival curve of the CX3CR1 and IFIT1 in TARGET. (C) Univariate Cox analyses for gene expression and clinical characteristics. (D) Nomogram constructed based on different prognostic variables. (E) Calibration Curve of the Nomogram Model.


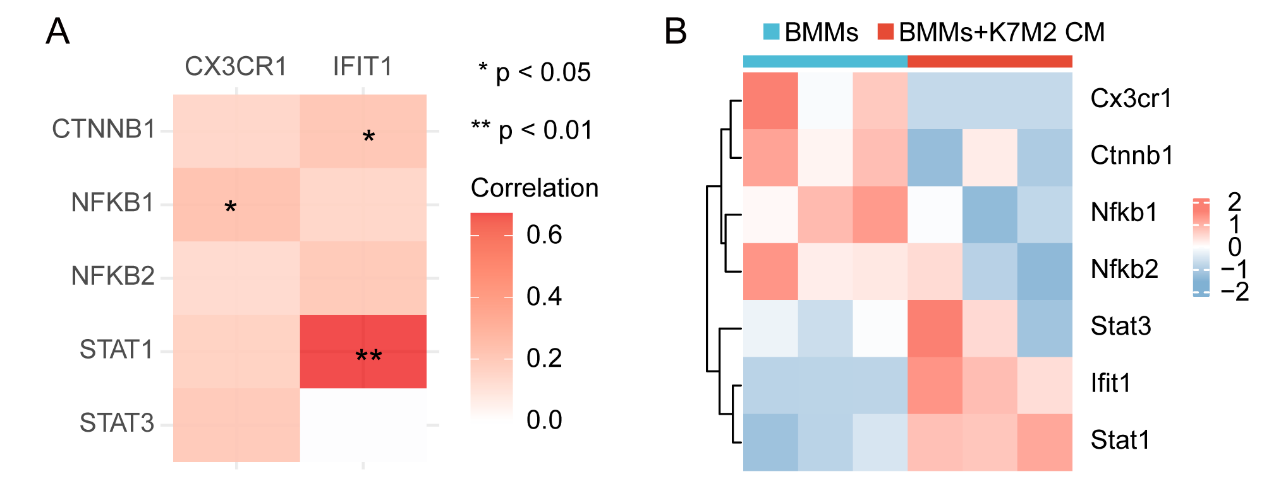


**Fig. S3. Potential key molecules mediating the function of IFIT1 and CR3CX1.** (A) Heatmap of gene-gene correlation

**Table S1. The information of the patient cohort from the RNA-seq data source.**

| **Patient** | **Sex** | **Age** | **Neoadjuvant chemotherapy** | **Tissue samples** | **Histological diagnosis** |
| --- | --- | --- | --- | --- | --- |
| 1 | Male | 16 | NO | Tumor tissue | Classical osteosarcoma |
| 2 | Female | 19 |  |  |  |
| 3 | Female | 45 |  |  |  |
| 4 | Male | 19 |  |  |  |
| 5 | Male | 14 |  |  |  |
| 6 | Male | 13 |  |  |  |
